# Attitude of Iraqi Rheumatologists While Managing Rheumatic Diseases in the Era of COVID-19: An Online Survey

**DOI:** 10.31138/mjr.31.3.299

**Published:** 2020-09-21

**Authors:** Nizar Abdulateef Jassim, Dina Yasiry, Yasameen Abbass Hummadi

**Affiliations:** 1 Rheumatology Unit, Department of Medicine, College of Medicine, University of Baghdad, Baghdad, Iraq; 2 Rheumatology Unit, Department of Medicine, Baghdad Teaching Hospital, Medical City, Baghdad, Iraq; 3 Department of Medicine, College of Medicine, Al-Nahrain University, Baghdad, Iraq

**Keywords:** COVID-19, Iraq, attitude, rheumatologist, survey

## Abstract

**Background:**

The current crisis COVID-19 has affected the already challenging management of rheumatic diseases. Since no evidence-based approach is yet available, this survey was conducted to explore the Iraqi rheumatologists’ attitude in the era of COVID-19 and how they have been managing their patients, especially with the continuously updating and sometimes conflicting reports.

**Patients and methods:**

A cross-sectional survey was distributed online via telephone and social media to rheumatologists in Iraq. A questionnaire was conducted about methods of patient education, preventive measures, and methods of arranging visits and treatment. In addition, an inquiry about the similarity of the practice between their local institutions and global ones.

**Results:**

144 rheumatologists answered the 14 obligatory questions, of which the majority were specialists. 122 rheumatologists participated in patient education. Half of participants used online information, social media and websites as a source to communicate and interact with their patients for education about COVID-19-related issues.

**Conclusion:**

Despite the lack of solid guidelines regarding the management of rheumatic diseases during the COVID-19 crisis, this survey showed the majority of Iraqi rheumatologists to be familiar with the updating recommendations. Also, as the majority are waiting for stronger evidence before attempting to embrace controversial issues, surely this reflects a responsible and scientific attitude.

## INTRODUCTION

Over the past few months, the world has been through a crisis described as the most influential in modern times. Coronavirus disease 2019 (COVID-19) has rapidly invaded the globe. The first recorded case of COVID-19 in Iraq was on 24^th^ February 2020. Until 8^th^ May 2020, the total number of confirmed cases reached 2,543 with, 102 deaths.^1^ The World Health Organization (WHO) praised Iraq for early endorsement of preventive measures, which has proven to be the only effective strategy so far, since no cure or vaccine is yet available.^2^ The management of patients with rheumatic diseases on immunosuppressants during the pandemic is very challenging. Such patients suffer an already dysregulated immune response, increasing the risk for a more severe infection. Health care workers themselves are at well-known risk for acquiring COVID-19 due to higher viral loads and repetitive exposures.^3^

Until now, no evidence-based approach in managing rheumatic diseases during the pandemic has been established. The published global recommendations^4–7^ are hindered by the lack of strong evidence and being influenced by experts’ opinions, which may not be suitable for different regions and health care systems.

With these facts in mind, this survey was conducted to understand rheumatologists’ attitude, and to facilitate making a unified local protocol for patients with rheumatic diseases in Iraq that is met with comfort among practicing physicians.

## METHODS

A questionnaire was formulated by the authors via Google Forms, submitted online and distributed by telephone, applications and social media on March 2020 for practicing Iraqi specialists and postgraduate students in the Rheumatology field.

The questionnaire had 14 obligatory single-choice questions: these included genders, work title, methods and opinions on patient education, preventive measures, scheduling visits, and if immunosuppressants and steroids were required. Questionnaire inquired which action was taken while COVID-19 contact or infection occurred, also prophylaxis for patients and health care provider. The last question was to be answered on a scale of 1 to 10 on similarity between the practices of the participants’ organisation and the international ones.

The obtained results are frequencies of answers, and are expressed as numbers and percentages of total. The results were analysed and obtained by Google Forms and Microsoft Excel software.

## RESULTS AND DISCUSSION

To the best of our knowledge, this is the first published survey from Iraq addressing the attitude of rheumatologists during COVID-19.

**Figure 1.**
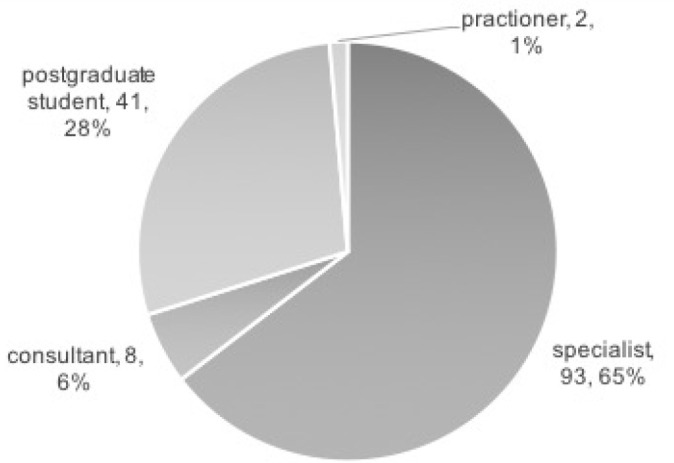
The current title of participants.

Of the 144 participants, 93 were specialists, 41 were postgraduate students. This reflects the excellent participation of students in academic projects, especially those related to hot topics like the current pandemic. The presence of only 2 practitioners highlights that Primary Health Care facilities are not optimal, and explains why most rheumatic patients insist on seeing a specialist,^8^ possibly due to drugs and lab shortage and the lack of updated knowledge.^9–11^ (**Figure 1**)

**Figure 2.**
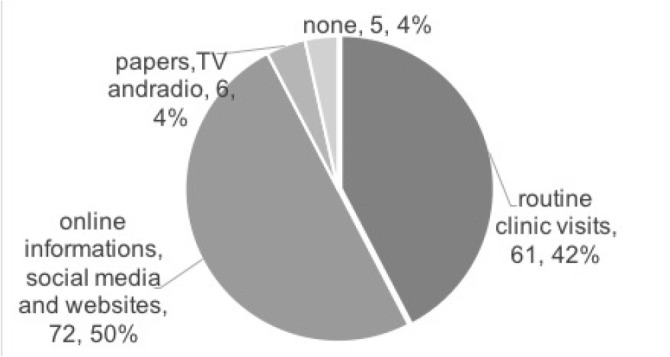
The distribution of the answers for the source of information in patient education.

**Figure 3.**
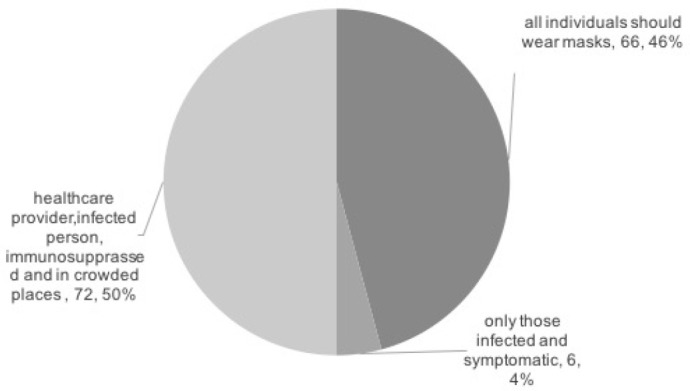
The distribution of the answers regarding the concept of wearing a mask.

Seventy-six rheumatologists (52.8%) were males. Involvement in patient education about COVID-19 and necessary preventive measures was reported in 122 participants (84.7%), which is highly expected, since such patients are at constant risk of infection. The other 22 (15.3%) stated that they haven’t been involved, and no reason is provided in the survey; however, they might had not been practicing due to curfew and closure of some clinics in certain cities, self-isolation for health-related reasons, being retired, or exclusive private sector work (**Table 1**). Since the answers were given anonymously, it was not possible to exclude those 22 participants from answering further questions. Seventy-two participants used online information, social media and websites to distribute information to their patients as a source of education. Other sources (**Figure 2**), eg, social media and internet, are expected to dominate as a source of communication and news in all age groups. Almost all hospitals and private clinics now have educating public Facebook and social media pages to interact with patients, and offer education and lifestyle advices. These pages were used from the beginning of the pandemic to spread knowledge about preventive methods. Many patients also communicate with their rheumatologists and postgraduate students through applications like WhatsApp, Viber, and others in their daily routine work to confirm schedules and review laboratory results. Luckily, this source was also used to communicate with patients. Seventy-two (50%) believed that health-care providers, the immunosuppressed, people in crowded places and infected individuals should wear masks, while 46% believed all individuals should wear masks as demonstrated in **Figure 3.** Actually, experts believe that stepping up the protection is guided by mask availability, and the different opinions come from variable recommendations. WHO recommends special masks for health-care workers working with proven/suspected COVID-19 cases.^12^ The Centre for Disease Control and Prevention (CDC) recently recommends everyone wear a cloth mask to prevent the spread, rather than protecting the wearer.^13^

**Table 1.** The results of participants’ answers to the questions of the survey.

**Variable**	**Results**	**144 (100%)**
Gender	Male	76 (52.8%)
	Female	68 (47.2%)
Educating patients with rheumatic diseases about the pandemic and the necessary preventive measures	Yes	122 (84.7%)
	No	22 (15.3%)
Do you think it is necessary to carry-on seeing your patients according to their pre-arranged appointments?	Yes	29 (20.1%)
	Routine visits must be cancelled, urgent complains are attended only.	104 (72.2%)
	Maybe	11 (7.6%)
Opinion about immunosuppressant use during the pandemic, given that neither infection nor a history of contact occurred?	Continue	127 (88.2%)
	Stop immunosuppressants	17 (11.8%)
Do you recommend starting patients on hydroxychloroquine as prophylaxis?	Yes	21 (14.6%)
	No	58 (58.3%)
	Not sure	39 (27.1%)
Do you recommend patients to receive routine vaccines as scheduled?	Yes, arrange vaccines according to timed schedule.	58 (40.3%)
	No, this can be delayed until the pandemic clears-off.	86 (59.7%)
Proved COVID-19 patient with Rheumatoid arthritis on Methotrexate, folate & prednisolone. what will your advice be about prednisolone?	Stop	42 (29.2%)
	Continue	60 (41.7%)
	Decrease the dose	42 (29.2%)
As a health care provider, have you been taking Hydroxy-chloroquine for prevention against COVID-19?	Yes, I am on a weekly dose.	25 (17.4%)
	No, I haven’t, not enough data.	116 (80.5%)
	I have a contraindication to it.	3 (2.1%)

One hundred four physicians believed that routine visits should be cancelled, and only urgent complains must be attended, which agrees with the global attitude^4–7^ as shown in **Table 1**.

When the participants asked about their attitude, in case no COVID-19 infection/contact had occurred, 127 (88.2%) rheumatologists stated that immunosuppressants are not to be stopped. This is similar to the global attitude; which shows a considerable interest and investment in the pandemic global knowledge.^14^ Eighty-four participants (58.3%) chose not to give their patients hydroxychloroquine as prophylaxis against COVID-19 (**Table 1**). This might be due to lack of compelling evidence, concern of shortage on Lupus patients and other indications.^(15)^ Still, the promising reports and its known safety encouraged 21 participants to use it. Eighty-six decided to delay routine vaccination, while 58 (40.3%) thought vaccinations should proceed as scheduled (**Table 1**). This difference is probably due to the pre-existing irregularities in the schedule due to the unfortunate interrupted supply of vaccination locally, which possibly led to the underestimation of its crucial importance. Another explanation is that some might think that deferring the vaccine would spare patients a hospital visitation during the pandemic.

**Figure 4.**
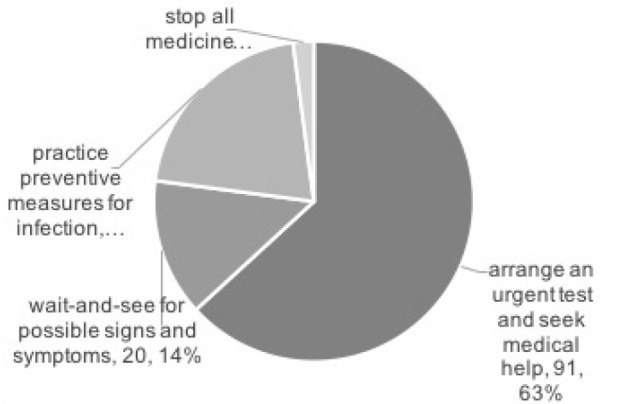
The participants’ answers regarding advice given to patients with contact with a documented COVID-19 case.

In case of contact with a documented COVID-19 patient, 63% of participants chose to advise their patients to arrange an urgent test and seek medical help, which is not the protocol indorsed by the WHO, especially if resources are not widely available.^16^ Twenty (14%) decided to wait-and-see, which is an acceptable approach, but definitely isn`t enough; 30 (21%) decided to advise for preventive measures, which might be reasonable to minimize community spread, but would not be enough or proper for the patient. (**Figure 4**)

**Figure 5.**
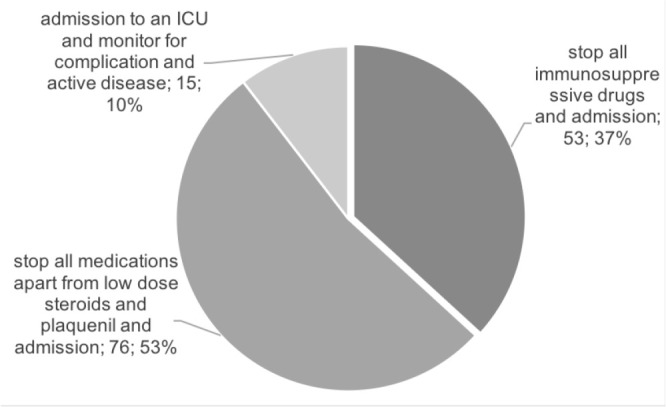
The appropriate action when a patient with rheumatic disease developed COVID-19.

In case a patient on immunosuppressive drugs was proved to be infected with COVID-19, 76 participants (53%) decided to stop all medication except for hydroxychloroquine and low dose of steroids;^17,18^ 53 (37%) stated they would stop all medication with admission; 15 (10%) answered with an ICU admission (**Figure 5**). Actually, a single/multiple answer(s) would apply here according to case characteristics and severity.

Another question was, if a patient with Rheumatoid arthritis on methotrexate, folate and 7.5mg prednisolone developed COVID-19. While inquiring about steroids status, sixty participants (41.7%) advised to continue prednisolone, while 42 (29.2%) chose to decrease dose. It is well known that chronic low dose steroid should not be stopped abruptly, but this can be a reflection of the conflicting reports.^17^ Eighty percent (116) of participants thought current data is not enough to support the use of hydroxychloroquine for COVID-19 prophylaxis, while 25 (17.4%) stated that they are already on a weekly dose, and 3 (2.1%) had a contraindication for the drug (**Table 1**).^19^

**Figure 6.**
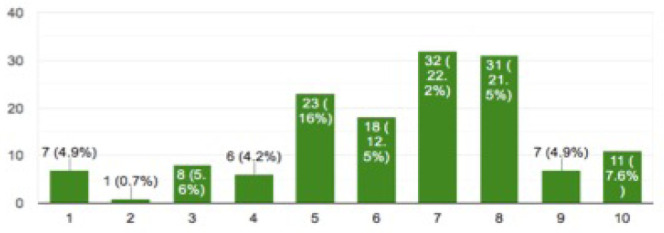
The distribution of 144 participants’ answers regarding their perception of similarity between their organizations’ protocols and global attitude.

Seven participants (4.9%) thought that their institution’s practice highly differs from the global attitude and assessed similarity to be 1/10, while 11 (7.6%) thought the similarity is 10/10. (**Figure 6**) Obviously, the majority believe their approach is close to that of global protocols because nearly 90% of the participants believed that the similarity is 5 or more, while only 10% believed otherwise.

## CONCLUSION

Despite that evidence-based global guidelines on how to manage rheumatic diseases during the COVID-19 pandemic are much needed, it is clear from the results of this survey that the majority of Iraqi rheumatologists are familiar with the continuously updating recommendations. Also, the majority are waiting for stronger evidence on controversial issues, which reflects a responsible and scientific attitude.
